# Hypophosphataemia in Critical Illness: A Narrative Review

**DOI:** 10.3390/jcm13237165

**Published:** 2024-11-26

**Authors:** Mahesh Ramanan, Alexis Tabah, Julia Affleck, Felicity Edwards, Kyle C. White, Antony Attokaran, Kevin Laupland

**Affiliations:** 1 Faculty of Health, Queensland University of Technology, Brisbane, QLD 4000, Australia; 2 Intensive Care Unit, Caboolture Hospital, Metro North Hospital and Health Services, Brisbane, QLD 4029, Australia; 3 Intensive Care Services, Royal Brisbane and Women’s Hospital, Metro North Hospital and Health Services, Brisbane, QLD 4029, Australia; 4 The George Institute for Global Health, University of New South Wales, Sydney, NSW 2000, Australia; 5 Intensive Care Unit, Redcliffe Hospital, Metro North Hospital and Health Services, Brisbane, QLD 4020, Australia; 6 Intensive Care Unit, Princess Alexandra Hospital, Metro South Hospital and Health Services, Brisbane, QLD 4102, Australia; 7 Intensive Care Unit, Rockhampton Hospital, Central Queensland Health Service, Rockhampton, QLD 4700, Australia; 8 School of Medicine, University of Queensland, St. Lucia, QLD 4072, Australia

**Keywords:** phosphate, intensive care, critical care, electrolyte, supplementation

## Abstract

Phosphate is a predominately intracellular anion that has several key roles in normal cellular functions. Derangements in serum phosphate concentration occur frequently during critical illness, particularly hypophosphataemia, which has been reported in up to 75% of Intensive Care Unit (ICU) patients. The association between hypophosphataemia and ICU outcomes reported in the literature are conflicting and and subject to substantial confounding. Exogenous phosphate can be administered in the ICU using the enteral and intravenous route safely. However, whether administering phosphate and correcting hypophosphataemia results in any patient-centred benefits, or harms, remains uncertain, particularly for patients with mild hypophosphataemia or low-normal phosphate levels. This review will highlight key aspects of hypophosphataemia management in the critically ill, summarise current best practice, and outline major research priorities.

## 1. Introduction

### 1.1. Physiology

Phosphate is a predominately intracellular anion that is essential for life. It is a critical component of nucleic acids, various structural proteins, phospholipids, the major cellular energy currency adenosine triphosphate, red blood cell 2,3-diphosphoglycerate, bones, and teeth [1]. Phosphorylation and de-phosphorylation are involved in key metabolic functions including energy storage and transfer, signal transduction of regulatory pathways, gene transcription, and enzyme catalysis [2].

In health, phosphate homeostasis is maintained by matching of dietary intake to urinary and faecal excretion and transcellular flux. Phosphate accounts for approximately 1% of body weight, with 85% of total body phosphate stored in bones and teeth (principally hydroxyapatite and crystalline calcium phosphate, respectively). Fourteen percent is found within cell membranes and in intracellular fluid, with only one percent found in extracellular fluid. Most extracellular phosphate (90%) is in the ionic form, with the remaining phosphate bound to proteins. Serum phosphate concentrations typically range between 0.80 to 1.50 mmol/L. Intracellular phosphate concentrations, in contrast, are typically 100–110 mmol/L1.

The endocrine control of phosphate (Table 1) is tightly bound to calcium homeostasis [3,4,5]. The serum phosphate and calcium concentrations are limited by the calcium–phosphate product, with calcium-phosphate precipitation occurring at calcium–phosphate product > 4.4 mmol^2^/L [2].

Parathyroid hormone (PTH) and calcitonin are the two main hormones that influence phosphate control. PTH secretion is stimulated by hypocalcaemia and results in phosphate release through osteolysis, reduces renal reabsorption of phosphate, and increases gastrointestinal absorption of dietary phosphate through stimulation of vitamin D3 production, which also increases renal reabsorption of phosphate negating the effect of PTH on the renal tubules. The net effect is mobilisation of phosphate stores and increase in serum phosphate concentration [3,4,5].

Calcitonin, stimulated by high serum ionised calcium, causes increased renal excretion of phosphate and stimulation of bone formation, leading to reduced serum phosphate concentrations [3,4].

Other hormones that influence control of phosphate, albeit at a lesser magnitude than calcitonin and PTH, include insulin, glucagon, corticosteroids, and the “phosphotonins”, of which fibroblast growth factor-23 (FGF-23) is the best characterised.

FGF-23 is secreted by osteocytes in response to serum phosphate levels and dietary intake. FGF-23 activity results in reduced gastrointestinal absorption and increased renal excretion of phosphate. It also suppresses PTH release from the parathyroid gland, leading to net loss of phosphate and reductions in serum phosphate concentrations [3].

### 1.2. Pathophysiology of Phosphate Derangements in Critical Care

Critical illness results in various derangements that can affect phosphate homeostasis, either directly or through derangements of serum ionised calcium [6,7,8]. Dietary intake of phosphate during critical illness is frequently absent, or sub-normal. Gastrointestinal absorption of phosphate and other nutrients is frequently diminished even when there is artificial nutrition provision. Renal excretion of phosphate can be augmented (e.g., by diuretics, which are commonly used in ICU) or reduced (by acute kidney injury or renal replacement therapy). Transcellular flux can be affected by exogenous insulin administration, commonly used for the treatment of critical illness-induced stress hyperglycaemia. Immobility can result in increased osteolysis, releasing phosphate from bone stores. Metabolic acidosis and alkalosis can have wide-ranging effects that can result in high or low serum phosphate concentrations. Sepsis, particularly with gram-negative bacteraemia and Legionella infections, can cause hypophosphataemia through uncertain mechanisms [9,10]. The refeeding syndrome, which arises due to pancreatic insulin secretion in response to caloric intake, can be associated with precipitous hypophosphataemia [11,12]. Disease processes such as extensive tissue injury (e.g., large surface-area burns, rhabdomyolysis) and tumour lysis syndrome can cause rapid release of phosphate and lead to hyperphosphataemia. Renal replacement therapy can also cause hypophosphataemia, though this can be mitigated by using phosphate-containing dialysis solutions [13].

Thus, phosphate derangements in ICU can be multifactorial and can result in either hypophosphataemia or hyperphosphataemia. As the critical illness evolves, both can occur at different stages.

## 2. Clinical Aspects

### 2.1. Incidence and Complications of Hypophosphataemia

The exact definition of hypophosphataemia is not universally agreed upon. A recent meta-analysis revealed that across twelve studies, thresholds of <0.48 mmol/L to <0.94 mmol/L were used to define hypophosphataemia [6]. Technical differences in laboratory practices may account for some of the variability in the definition of hypophosphataemia. However, a nearly two-fold variation in the threshold for hypophosphataemia suggests substantial clinical uncertainty among clinicians. Mild degrees of hypophosphataemia are generally well tolerated without any adverse symptoms [14].

Hypophosphataemia has been reported to occur in 10–75% of critically ill patients managed in ICUs [6,7,8,15,16,17], though as noted, the reported incidence is dependent on the definition of hypophosphataemia. In the largest of these studies, a multicentre cohort study with 77,310 patients from twelve ICUs over a six-year period, 34% of patients developed hypophosphataemia using a threshold of <0.80 mmol/L, mostly on day 2 of ICU admission [15].

Severe hypophosphataemia, which also does not have a consistent definition, is believed to cause serious adverse effects such as rhabdomyolysis, skeletal muscle weakness, respiratory insufficiency, cardiac rhythm disturbances, peripheral neuropathy, haemolysis, delirium, seizures, and coma. The exact threshold below which these complications may occur, and the extent to which these can be attributed to low phosphate levels, is not known. However, for practical purposes, we recommend that serum phosphate concentration <0.30 mmol/L should be considered severe hypophosphataemia and be treated to avoid potential complications. Severe hypophosphataemia, using a threshold of <0.30 mmol/L, is much rarer, observed in only 0.8% of patients in the aforementioned large multicentre cohort study [15]. While there is no broad consensus on this matter, at the authors’ institutions, moderate hypophosphataemia is defined as serum phosphate concentrations of 0.30–0.49 mmol/L. There is currently also no consensus on the threshold for mild hypophosphataemia, and the clinical significance of phosphate concentrations between 0.50–0.79 mmol/L is unknown. It is possible that phosphate concentrations in this range may represent a “low-normal” physiological state.

Hypophosphataemia has been associated, albeit inconsistently, with various adverse clinical outcomes such as increased mortality and increased ICU and hospital length of stay. A meta-analysis of 12 studies comprising 7626 patients concluded that hypophosphataemia among ICU patients was associated with prolonged ICU and hospital length of stay, but not increased mortality, and that it may be a marker of illness severity [6]. An observational study [18] demonstrated that even severe hypophosphataemia (with thresholds of <0.20 and <0.30 mmol/L tested) was not associated with increased mortality after adjustment for illness severity. This study concluded that hypophosphataemia is a marker of illness severity, rather than an independent risk factor for adverse outcomes. Conversely, other multicentre observational studies found that even after adjustment for illness severity, mechanical ventilation, treatment limitations, diagnostic group, and age, hypophosphataemia remained a significant independent predictor of increased mortality and ICU length of stay [15,17].

Therefore, hypophosphataemia is common among ICU patients and may be associated with worse clinical outcomes, though the evidence for such associations is conflicting. On the other hand, it may simply be an epiphenomenon of critical illness and be a marker of illness severity. Thus, it is possible that benefits from aggressive phosphate replacement may be non-existent, minimal, or even harmful. There is currently no randomised trial evidence that shows benefit from phosphate replacement in asymptomatic patients in the ICU [19,20].

### 2.2. Exogenous Phosphate Replacement

Hypophosphataemia is managed in the ICU by identification and treatment of the cause and pharmaceutical replacement of phosphate. Causes such as refeeding syndrome, high-dose insulin infusion, reduced oral intake, metabolic alkalosis, and diuretic therapy all have specific management options [11,19,21]. These may include provision and modification of artificial nutritional input (specifically decreasing caloric delivery), glycaemic targets, ventilatory management (for hypercarbia-driven metabolic alkalosis), and modification of diuretic strategy.

Replacement, or exogenous administration, of phosphate can occur through three means—oral nutritional intake, artificial nutrition delivery (either enteral or parenteral), and pharmaceutical administration either enteral or intravenous [14,19]. Nutrition in ICU is typically titrated to estimated caloric and protein requirements. It is not titrated to serum phosphate, and as such, nutrition provision has a limited role in management of hypophosphataemia, with the major exception of refeeding syndrome, which would require reduction and tightly controlled uptitration of caloric delivery.

Enterally, phosphate can be administered as sodium phosphate monobasic tablets. Each tablet typically (exact preparations vary globally) contains 500 mg of elemental phosphate (16.1 mmol of phosphate anions) and can be crushed for administration via a naso- or oro-gastric tube for patients who cannot swallow. Monobasic sodium phosphate is also available in effervescent tablets. The bioavailability of enteral phosphate is 70% [22]. While generally well tolerated, enteral phosphate preparations can occasionally cause nausea, vomiting, and diarrhoea, limiting their use in critically ill patients.

Several intravenous phosphate preparations are available globally. Monobasic sodium phosphate 15.6% solution is available in 10 mL ampoules containing 10mmol of phosphate anions. One ampoule is typically diluted in either 100 mL (for central venous administration) or 250 mL (for peripheral administration) of 0.9% sodium chloride solution and infused over 4–6 h as per facility-specific guidelines. Faster rates can be administered safely if clinically required [23]. Potassium dihydrogen phosphate 13.6% solution also contains 10 mmol of phosphate anions and is administered in a similar manner to monobasic sodium phosphate, though greater care must be taken to prevent rapid administration due to its potassium content. Some healthcare jurisdictions, such as the authors’ institutions, have stopped stocking potassium dihydrogen phosphate to eliminate the possibility of harm from inadvertent rapid potassium infusion through the administration of this potassium-containing phosphate preparation.

Inadvertent rapid administration of intravenous phosphate can lead to widespread intravascular calcium precipitation [19]. Severe ionised hypocalcaemia with resultant tetany, severe neuromuscular weakness, seizures, and coagulopathy may ensue. Other complications can include hyperphosphataemia, hypomagnesaemia, and hypotension [24,25,26]. The risk of these complications occurring may be avoided or reduced by judicious and slow replacement of phosphate.

Given its high bioavailability and safety profile, enteral phosphate should generally be used preferentially whenever possible for critically ill patients. An observational study [27] reported that during a period of national shortage in the Unites States of America of intravenous phosphate (i.e., only enteral phosphate available), there was no increase in duration of mechanical ventilation or mortality, compared to a control period when both formulations were available. A recent non-inferiority randomised trial concluded that enteral phosphate replacement was non-inferior to intravenous replacement [28]. Thus, given the serious potential for harm with intravenous phosphate, where phosphate replacement is thought to be clinically indicated, a strategy of preferentially using enteral phosphate preparations should be adopted.

### 2.3. Current Recommendation and Clinical Practices

With the lack of evidence of benefit [29,30,31] to guide phosphate replacement in the ICU, current clinical practices are highly variable [15]. While guidelines for management of hypophosphataemia at international [32], national [33,34], and local [35,36,37] levels do exist, their contents are variable. Current recommendations are based on expert opinion and weak evidence from retrospective studies [20]. As such, there is an immense clinical need for generating new evidence to guide practices in this regard.

In general, intravenous phosphate replacement is recommended for severe hypophosphataemia, with a threshold of ~0.30 mmol/L, though it must be recognized, and emphasized that the patient-centred benefits of such a strategy is unproven [20,38]. Recommendations for mild-moderate degrees of hypophosphataemia with serum concentrations between 0.30 and 0.80 mmol/L are more variable. Conservative management with observation and monitoring together with removal of causative factors where possible, or enteral replacement of phosphate, have both been recommended without any evidence to guide a strong preference for either approach. Phosphate concentrations between 0.50 mmol/L and 0.79 mmol/L may represent a low-normal physiological state in response to critical illness and may not require any specific treatment.

This general uncertainty is reflected in bedside clinical practice with one practice survey demonstrating that only 23/56 (41%) ICUs had specific unit recommendations for phosphate replacement [36]. A recent multicentre observational study demonstrated marked variability in the thresholds at which phosphate was replaced across twelve ICUs in the state of Queensland, Australia [15]. In this study, patients with mild hypophosphataemia (serum concentration 0.50–0.79 mmol/L) had a markedly variable chance of receiving phosphate replacement ranging from 25% to over 80% depending on which ICU they were admitted to. Whilst almost 100% of patients with severe hypophosphataemia (<0.30 mmol/L) received phosphate replacement, even among patients with moderate hypophosphataemia (0.30–0.49 mmol/L), there was some variability with 70% to almost 100% of patients receiving phosphate at different ICUs. Wide variation in the relative usage of intravenous and enteral phosphate preparations was also observed in this study.

## 3. Research Considerations

### 3.1. Evidence Gaps and Future Research (Table 2)

Hypophosphataemia is a commonly encountered phenomenon in ICUs of uncertain significance for patients. The evidence is conflicting as to whether hypophosphataemia is simply a marker of severe critical illness or whether it is causally implicated in worsening outcomes. Severe hypophosphataemia can be associated with life-threatening complications such as arrhythmias, rhabdomyolysis, seizures, and coma, and thus should be promptly treated with phosphate replacement. While the intravenous route may be appealing due to potential for rapid correction, enteral phosphate administration is effective, safe, inexpensive, and should be strongly considered in most circumstances.

For patients with mild and moderate hypophosphataemia, substantial uncertainty in phosphate management exists. High-quality, prospective, longitudinal, observational studies are required to determine whether there is an independent causal link between hypophosphataemia and worse outcomes, such as mortality, prolonged ICU or hospital length of stay, prolonged mechanical ventilation duration, and worse long-term, functional outcomes. Due to selection bias that is rife in most of the existing literature, prospective studies with few exclusion criteria, and detailed observations of patients’ physiology, biochemistry, clinical status, and outcomes are required. There may be a role for embedding such studies within electronic medical records and quality assurance initiatives as it is likely that large sample sizes will be required to detect the small differences in outcomes that can be plausibly attributed to phosphate abnormalities.

A particular area of research that requires further attention is defining the threshold that differentiates low-normal phosphate levels from hypophosphataemia. Current practice is based upon arbitrary cut-offs derived from laboratory reference ranges. However, the threshold below which patient-important harm starts to occur should ideally be used to define the level below which hypophosphataemia is said to be present. Following on from this, mild, moderate, and severe hypophosphataemia could be objectively redefined on the basis of unbiased associations with patient outcomes.

Further, the optimum management of hypophosphataemia needs to be studied in rigorous clinical trials. Hypophosphataemia management may include the following components: addressing the underlying cause where relevant (e.g., refeeding syndrome, insulin infusion, diuretic therapy), necessity of phosphate replacement (particularly for mild hypophosphataemia or low-normal phosphate levels), replacing phosphate through dietary sources versus pharmaceutical supplementation, intravenous or enteral phosphate, threshold at which to commence phosphate replacement, and monitoring frequency. Currently, only one of these topics has been studied in randomised trials—intravenous versus enteral phosphate replacement. Most current evidence suggests that enteral replacement is as effective as intravenous [28], and thus intravenous phosphate should be reserved for emergencies, patients who cannot tolerate any enteral input, and possibly for clinical trial scenarios.

At a more basic level, whether phosphate should be routinely monitored or replaced at all (unless patients have attributable symptoms from hypophosphataemia) should be studied. It is quite plausible that the current practices of replacing phosphate based on low-quality evidence is either unnecessary or, in the worst-case scenario, harmful. Other attempts to normalize abnormal parameters in critical illness, such as glucose and cardiac output, have proven to be ultimately futile or harmful [39,40,41,42,43,44]. Decades of research aimed at strictly normalizing glucose levels in the ICU were eventually shown to be non-beneficial and harmful in some patient populations. Early goal-directed therapy for sepsis, which included cardiac output augmentation, followed a similar trajectory, where initial purported benefits were disproven by subsequent large, randomised multicentre trials. It is currently entirely possible that phosphate management in critical illness could follow a similar path, one that should be avoided with a targeted research agenda.

**Table 2:** Key evidence gaps.

Evidence Gap	Potential Question/s
Monitoring of phosphate levels in ICU	Is daily monitoring necessary? Or beneficial? Or harmful?
Significance of hypophosphataemia in critical illness	Is hypophosphataemia a marker of illness severity? Is it associated with clinical outcomes? Any patient-important harms?
Threshold for defining hypophosphataemia	0.80 mmol/L or lower? At what level does harm ensue?
Severity of hypophosphataemia	Cut-offs for mild, moderate, and severe, preferably based on objective evidence of harms
Treatment thresholds	At what phosphate level should phosphate replacement be initiated?
Overall management of phosphate	Holistic management of phosphate including monitoring, nutrition, and supplementations. Should phosphate management algorithms be devised and tested?

### 3.2. Currently Recruiting Studies

Some currently recruiting studies may shed further light on phosphate management during critical illness.

A prospective, observational study from the United Kingdom may provide further data on institutional practices with regards to phosphate management and large-volume epidemiological data to investigate the link between hypophosphataemia and patient outcomes (clinicaltrials.gov ID: NCT06376461). This study may produce important epidemiological data that could help redefine thresholds of hypophosphataemia.

A phase-2, cluster-crossover, randomised trial comparing two different phosphate thresholds (0.80 mmol/L and 0.50 mmol/L) for the initiation of phosphate replacement may produce evidence to guide phosphate therapy in the ICU (Australia New Zealand Clinical Trials Registry ID: ACTRN12622001193763). Specifically, it may help answer the question of whether treatment of phosphate levels between 0.50–0.79 mmol/L is beneficial.

A single centre randomised trial comparing intravenous with enteral phosphate replacement for hypophosphataemic patients in the ICU may inform clinicians’ decision on the route of administration of phosphate (Iranian Clinical Trials Registry ID: IRCT20150107020592N18).

A search of the World Health Organization’s clinical trials platform and clinicaltrials.gov did not reveal any other registered currently recruiting studies. While this may not exclude the possibility of other studies being conducted, the current number and type of studies are insufficient to answer all the outstanding questions with regards to hypophosphataemia management in critical illness.

## 4. Conclusions

Hypophosphataemia in critical illness is common and has uncertain associations with patient outcomes, and current evidence is insufficient to guide optimum management. Phosphate can be effectively replaced with either intravenous or enteral supplementation, but substantial uncertainty exists as to the threshold at which phosphate should be replaced in the ICU.

## Figures and Tables

**Table 1:** Factors effecting serum phosphate concentration.

	GIT Absorption	Renal Reabsorption	Osteolysis	Bone Formation	Intracellular Shift	Net Effect On Serum Phosphate
PTH	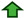	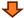	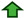	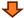		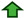
Vitamin D3	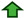	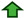	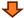	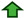		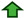
Calcitonin		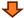	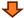	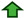		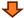
FGF-23	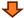	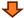				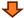
Insulin					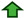	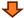
Glucagon					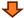	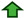
Corticosteroids		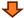				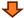
